# An attention approach to emoji focused sarcasm detection

**DOI:** 10.1016/j.heliyon.2024.e36398

**Published:** 2024-08-21

**Authors:** Vandita Grover, Hema Banati

**Affiliations:** a Department of Computer Science, University of Delhi, India; b Dyal Singh College, University of Delhi, India

## Abstract

Emojis play a nuanced role in digital communication and have a potential to convey sarcastic intent as they often offer non-explicit and sometimes ambiguous cues. This ambiguity has a potential to fuel hate-speech, trolling, or cyber-bullying under the guise of sarcasm. There have been numerous studies that employ modalities like audio, images, videos, emojis or a combination of modalities to detect sarcasm in online text. There is limited research that focuses solely on the impact of emojis in discerning sarcasm. Therefore, in this work we use popular attention networks to capture if sarcasm classification can be improved when emojis are present in text. We experiment with LSTM, Bi-LSTM, and attention networks and compare the results with the fine-tuned benchmark DeepMoji model. Our experiments demonstrate that the emojis can help improve sarcasm classification. These models outperform the benchmark DeepMoji model on two different test datasets on Matthew's correlation coefficient and Area under the curve metrics. Our proposed models surpass DeepMoji by an increase in **0.22** and **0.25** when compared for MCC and an increase in **13.3 %** and **14.76 %** for the ROC-AUC metric.

## 1 Introduction

The Oxford Dictionary describes sarcasm as, “A sharp, bitter, or cutting expression or remark; a bitter gibe or taunt” [1]. It is this bitterness or aggressive intent that makes sarcasm a tool for hurtful language [2]. Hate speech becomes a more complicated problem to solve as sarcastic content may not be explicit [[3], [4], [5]]. This makes sarcasm detection one of the most important tasks that attracts the focus of several researchers.

There have been several modalities that have been exploited to enhance sarcasm detection tasks in online text, which include, figurative speech, linguistic features, images, audio, video, hashtags, and a combination of modalities among many others.

Emojis as a modality for sarcasm detection tasks too has gained traction over the past few years. But emojis are used as one among many other modalities to improve the classification accuracy or F1-score of sarcasm classifiers. We are yet to come across studies that exclusively study how addition of emojis to text impact sarcasm classification.

But why emojis?

Every emoji has a specific Unicode according to the Unicode Consortium that helps in encoding emojis for the machines. For example,  has a Unicode U+1F644 [6]. The Unicode website also lists resources for information and references on Emojis, with Emojipedia (a member of the Unicode Consortium) as a resource for information for a specific emoji. Emojipedia describes an emoji in text along with common interpretation. For example,  is used to convey disdain, boredom, disapproval, or frustration [7]. This emoji could be used with varying tones like sassy, resentment, sarcasm, or playful.

It may happen that a user interprets an emoji differently from the popular usage given on Emojipedia or other emoji resources. This difference in interpretation of emojis by different users may alter the interpretation of the message the emoji(s) has been used with. This has been discussed in our work [8,9]. Hence, the experiments presented in this work are important as they focus on emoji usage by different users in sarcastic and non-sarcastic texts. The distribution of emojis w.r.t. sarcastic and non-sarcastic texts is reported in Ref. [8].

With this work we experiment with different deep learning networks including attention mechanisms to study if introduction of emojis to text helps in improving sarcasm detection.

When analyzing classification performance of classifiers, usually F1 is the preferred metrics used by researchers. Recently researchers have identified Matthews correlation coefficient (MCC) and Area under the Curve (AUC) to be more discriminating than F1-score [[10], [11], [12]]. Therefore, in this study we will use F1, MCC, and AUC to compare classification performance.

For this study we pose the following research questions.

1. Does the addition of emoji(s) to text helps improve a sarcasm classifier performance?
2. Can the most repeated (the most frequent) emoji occurring in text can help in sarcasm detection?
3. Is it sufficient to use F1-metric to evaluate a binary classifier's performance?

This research paper is organized as follows. In the Related Work section, we discuss the ongoing research that incorporates emojis for sarcasm classification. The Methodology section describes several deep-learning networks including attention mechanisms created to quantify the impact of emojis in sarcasm classification. We also compare the performance of these networks with the benchmark DeepMoji model [13]. The findings are presented in the Experimental Results section. In the Discussion section we compare the performance of the proposed models with the benchmark DeepMoji model. The Conclusion and Future Work section concludes our findings and presents the direction for future research.

## 2 Related work

While sarcasm detection has several published research works [[14], [15], [16], [17], [18], [19], [20]], our focal point is the use of emojis for sarcasm detection. There have been many research works that use deep learning architectures to work with emojis in sarcasm detection tasks, some of which are discussed as follows.

Recently, a hybrid deep learning model [21] which is combination of sequential CNN followed by LSTM layers was used for sarcasm classification in Hindi tweets using word and emoji embeddings for sarcasm detection in Hindi tweets and Sarc-H dataset (1004 records). The authors report an improvement of 7 % in with F1-score on the test when emojis were used with text.

An emoji aware deep learning framework is proposed for multimodal sarcasm detection [22]. The SEEmoji MUStARD dataset (3641 records) was created by manually annotating the multimodal MUStARD dataset [23]. The MUStARD dataset has audio-visual conversational dialogues from four famous TV shows which have been labeled with a single emoji and corresponding sentiment and emotion. The authors use a bidirectional Gated Recurrent Unit with word, video, and acoustic utterances as input and an emoji as label. Several combinations of modalities are experimented with to obtain different emoji-aware multimodal representations. A Gated Multimodal Attention (GMA) is employed for sarcasm detection. The best F1-score of 76.7 % for speaker dependent utterances and 69.8 % for speaker independent occurrences was achieved in this experimental work. This was achieved using 5-fold cross validation when all the other modalities (viz. text, visual, and audio) were present.

The TANA architecture [24], uses word and emoji embeddings to leverage both sequential and non-sequential information for sarcasm classification in resource poor Indian indigenous language. In this work utilizes pre-trained fast-text Hindi word embeddings and emoji2vec embeddings for emojis are utilized. LSTM model with a squared hinge loss function is used to train the classifier to achieve F1-score of 96.75 % on the test set.

The Deepmoji model [13] use millions of emoji occurrences to learn emotional word representations and classify emotional content of text. The authors label each text with an emoji label (from the emoji in the text) and train a classifier for emoji classification task. This classifier uses two Bi-LSTM layers and attention layer [25,26] followed by a softmax layer for classification. This pre-trained model can then be fine-tuned using three approaches and a transfer learning approach (details in Section 3) on three NLP tasks viz. sentiment, emotion, and sarcasm detection using eight datasets. For sarcasm detection they achieve best results with 69 % F1-score on SCv1 dataset (1995 records) [27] and 75 % F1-Score on SCv2-GEN dataset (3260 records) [28] with the chain-thaw approach.

In the reported research work, the datasets used for training [21,22,24] and validation [13] are small and experimental results are usually reported on the validation datasets. For experiments presented in this work we use SarcOji dataset for training which has 29377 records [8]. We also use curate two different datasets with 7228 and 5056 records for testing our models’ performance.

Many researchers focus on accuracy or F1-score to report performance of the classifiers. Also, the core focus of many research work is architectures and emojis are merely used as one of the modalities to support sarcasm classification. With this work we aim to study if employing emojis with text can help improve sarcasm classification, in absence of any other modality. We present the experimental results and report classifiers’ performance using three different metrics.

## 3 Methodology

For this work we have employed different deep-learning architectures to understand the impact of emojis in sarcasm detection task. We use architectures like a vanilla sequential model, LSTM, and Bi-LSTM networks.

We also experiment with two popular attention approaches for our experiments that are described next.

### 3.1 Attention network

**Fig. 1:**
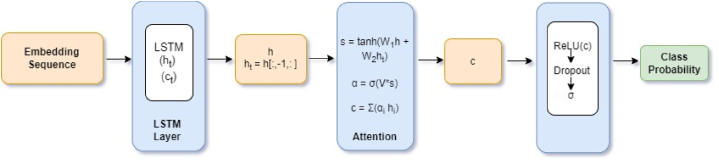
– Additive attention architecture block diagram [25],

Bahdanau et al. additive attention [25] is one of the most common attention mechanisms in sequence-to-sequence tasks. For our work we use the Bahdanau et al. approach for our sentiment classification task by applying a sigmoid activation to class probabilities of sarcastic and non-sarcastic classes. We call this approach additive attention for simplicity. Fig. 1 depicts a block diagram for additive attention for sequence classification.

The input layer accepts a sequence of fixed length inputs.

$$x=\left(x_{1},x_{2}\ldots ,x_{T}\right)$$

Where T is the maximum sequence length.

The embedding layer captures the vector representation (embedding sequence) of text from the embeddings.

$$e=\left(e_{1},e_{2}\ldots ,e_{T}\right)$$

e_i_ = E(x_i_) for embedding matrix E ∈ R^V^ ^X^ ^D^

Where V is vocabulary size and D is dimension of the embeddings (in our case 300).

The additive attention mechanism takes hidden state (h_t_) and last hidden state (h_t_[:,-1,: ]) as inputs and calculates attention scores to focus on the most relevant parts of inputs to produce the context vector (c).

The score calculation at time stamp t is given as:

$$s=\tanh\left(W_{1}h+W_{2}h_{t}\right)$$

where, W1 and W2 are learnable weight matrices and h_t_ is hidden state at time stamp t.

Attention weights are computed as

α=σ(V*s)

V ∈ R^units^ ^X^ ^1^ transforms the score followed by softmax normalization to obtain attention weights α (- for each element at time stamp t).

The context vector C is computed

$$c=\sum \alpha_{i}*h_{i}$$

The attention weight of each element (importance) is combined with the hidden state of the element (information captured about element) and contribution of all elements are added together to generate the context vector. This context vector is then passed through a dense layer with ReLU and then a dropout layer to avoid overfitting. The last (output) layer employs the sigmoid function for our sarcasm classification task.

**Fig. 2:**
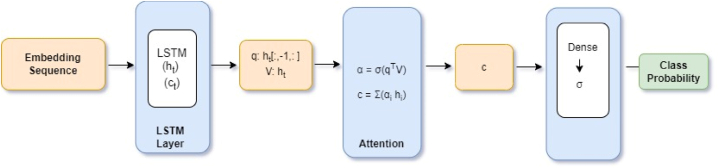
– Multiplicative attention architecture block diagram [29],

Luong et al. too proposed three attention approaches, viz. using dot product of encoder and decoder states, concatenation of decoder states and local attention that uses a window mechanism to focus on a localized subset of encoder states, [29]. For our work we use Luong's dot product attention mechanism and call it Multiplicative attention (Fig. 2). We use sigmoid activation function to find class probabilities for the binary sequence classification task of sarcasm detection for our work.

The vector representation captured by the embedding layer is passed to LSTM network. LSTM is applied at each time step i to the embedding vector, h_t_ and c_t_ are the hidden and cell states respectively.

$$h_{t},c_{t}=\text{LSTM}\left(e_{i},h_{t-1},c_{t-1}\right)$$

The final hidden LSTM layer is the query vector q that is the focus of the decoder in present state.

q is the last hidden state of the LSTM at time stamp t and V the value vector output from the LSTM layer, containing hidden states for each time step. $s=q^{T}V$.

Values V contain the representation of each element in the sequence.

The dot product between q and V measures similarity between decoder focus(q) and the relevant information captured from the input sequence (values).

Attention weights are normalized, α.

α=σ(s).

The context vector c is computed as $c=\sum_{i}\alpha_{i}h_{i}$.

The context vector is then passed through a dense layer and sigmoid is applied to achieve the final prediction for sarcasm classification.

Next, we discuss the proposed models that employs emojis in text for sarcasm detection.

### 3.2 Proposed Work

For this research work we use the SarcOji dataset [30]. This dataset contains 29377 texts all with emojis with 11448 records labeled as sarcastic and 17929 records labeled as non-sarcastic. Apart from several derived features in this dataset we focus on the MaxEmoji column in this dataset. MaxEmoji is the emoji occurring the most in the text. For instance, if the text is “ You are so funny   ”. Then MaxEmoji column in the SarcOji dataset will have  for this text. The text with only one emoji will have the said emoji in the MaxEmoji column. For example, if the text is “You are so funny ” will have  in the MaxEmoji column. In case of text that have multiple emojis each occuring once in the text, the first emoji is considered as the MaxEmoji. This is discussed in the algorithm Frequent Emoji Position and Intensity [30].

It was observed in several studies [[31], [32], [33], [34]], and our previous work [30,35] that emoji usage differs among users and may be different from the visual cues they offer or the intended meaning conveyed by the emoji description.

We hypothesize that if a user employs a single emoji or repeats an emoji in text, they want to convey a strong emoji. Thus, it is possible that the most frequent emoji (MaxEmoji) may be able to capture the underlying sarcasm in text. Hence, this work employs sarcasm-aware emoji embeddings and attention mechanism to enable machines detect sarcasm, even if the emojis are used differently than their original intended meaning by the users.

### 3.3 Embeddings

For the experiments in this work, we have used GloVe embeddings [36] for text.

**Fig. 3:**
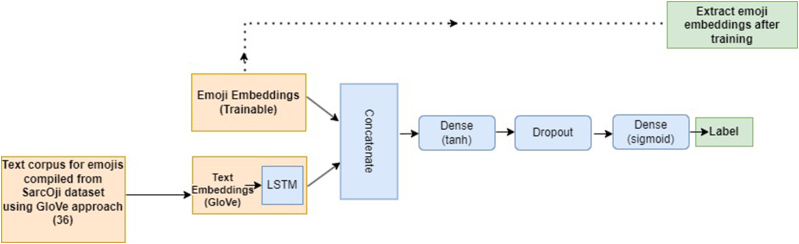
– Sarcasm aware GloVe emoji embeddings.

For emojis we create emoji embeddings from the text corpus from the SarcOji dataset. We use the methodology employed in GloVe: Global vectors for word representation [36], and combine text that occurs with different emojis in the dataset. We then train a pseudo classifier for sarcasm detection task and extract emoji embeddings that are learned in the process. Fig. 3 demonstrates the block diagram to demonstrate the emoji embedding process.

We call these emoji embeddings as sarcasm-aware GloVe based emoji embeddings.

Both the word and emoji embeddings length are in 300-dimensional space.

### 3.4 Proposed models

We test and compare three models that we run on different deep-network architectures to compare how the inclusion of emojis impacts performance of sarcasm classifiers. The PlainText model is used as a baseline to compare TextWithEmojis and TextMaxEmoji models proposed in this work, The models are listed as follows.

1. Model 1: Text only. This is a baseline model which consists of only text data. In this model we strip the text in the dataset of all the emojis so that only the words remain. We call this model *PlainText model*.
2. Model 2: Text and Emojis. This model uses the text with emojis as they originally occurred with the text in the dataset. We call this model *TextWithEmojis model*.
3. Model 3: Text and MaxEmoji. In this model we strip the text off all the emojis and append the MaxEmoji at the end. We call this model *TextMaxEmoji model.*

Next, we describe the architectures used to test the proposed models.

### 3.5 Deep-layered architectures and hyperparameters

We experiment these models on several deep network architectures listed as follows.

1. Vanilla sequential model containing a linear stack of layers
2. LSTM
3. Bi-LSTM
4. Additive attention mechanism (Bhadnau's additive attention adapted for this work)
5. Multiplicative attention approach (Luong's dot attention)

We conducted a Grid Search [37] for determining the hyper-parameters for the attention mechanisms. The following hyperparameters were found to be optimal and were hence used for all the architectures.

• Dense units: 8
• Dropout: 0.02
• Optimizer: Adam
• Learning rate: 0.01
• Activation function: Sigmoid at the final layer

A batch size of 32 with 25 epochs and an early stopping criterion of 6 epochs was used for all the experiments. We experimented with 8, 16, and 32 attention units. The architecture with 16 and 32 attention units gave similar results. These results were better than the architecture with 8 attention units. We thus chose 16 attention units as a balance between performance and network complexity.

### 3.6 Fine tuning from pre-trained DeepMoji model for sarcasm detection

As discussed in the Related work section, the DeepMoji [13] uses millions of occurrences for pretraining models for emotion related target tasks and has a diverse emoji set. This model will serve as an ideal comparison benchmark to compare our proposed models' performance for emoji focused sarcasm detection. We use DeepMoji's pretrained model and fine-tune it on the SarcOji dataset for sarcasm detection. We use all the four approaches described in DeepMoji to compare our model performances. The fine-tuning approaches for the target task (in this case sarcasm detection) are listed as follows.

• DeepMoji(last): All layers except the ‘last’ layer are frozen when fine-tuning.
• DeepMoji(full): All layers are unfrozen when fine-tuning.
• DeepMoji(chain-thaw): Layers are sequentially unfrozen and fine-tuned sequentially
• DeepMoji(new): No pretraining.

### 3.7 Test datasets

Most research work we have come across report the experimental results on the validation dataset. For generalization of our findings, we report the results on two unseen datasets. SarcOjiTest1 has been compiled from the benchmark datasets [[38], [39], [40]]. While for SarcOjiTest2, we scraped random tweets from Twitter (now X), posted in years 2021 and 2022. We used hashtags like #sarcastic, #sarcasm, #whatever, #not, #lmao, #sarcasmic, #sarcasticmemes, #lmao, #wtf for gathering sarcastic tweets. While the non-sarcastic tweets were scraped where posts did not include these hashtags. The details of the test datasets are reported as follows (Table 1). Both the test datasets have been compiled using the methodology used for SarcOji dataset [30].

**Table 1:** Datasets Statistics.

Dataset	Sources	Sarcastic (%)	Non-sarcastic (%)	Texts with 1 emoji (%)	Texts with more than 1 emoji (%)	Total texts
SarcOji	[30]	38.97	61.03	69.82	30.18	29377
SarcOjiTest1	[[38], [39], [40]]	52.6	47.54	56.48	43.51	7228
SarcOjiTest2	Tweets scraped from Twitter (now X)	25	75	53.09	46.91	5056

The distribution of emojis, emoji usage in text, and usage pattern of the most occurring emojis in the SarcOji dataset are discussed in the work on curation of SarcOji dataset [30].

For performance of different models across different architectures we use F1-score, Matthew Correlation Coefficient (MCC), and Receiver Operating Characteristic Curve – Area Under the Curve (ROC – AUC) score as comparison metrics discussed next.

### 3.8 Evaluation metrics

F1-score is metric of choice to compare a binary classifier's performance.

F1 is computed as:

F1=TPTP+12(FP+FN)

MCC can be computed as:

$$MCC=\frac{TN*TP-FN*FP}{\sqrt{\left(TP+FP\right)\left(TP+FN\right)\left(TN+FP\right)\left(TN+FN\right)}}$$

MCC ranges from −1 to 1. A higher correlation means the model predicts both the classes accurately. If MCC is 0 that means the model is not able to distinguish between both the classes. A negative MCC score means the model is predicting the opposite of what it is supposed to predict or flipped predictions.

Where, TP = True Positives, TN = True Negatives, FP = False Positives and FN = False Negatives.

**Fig. 4:**
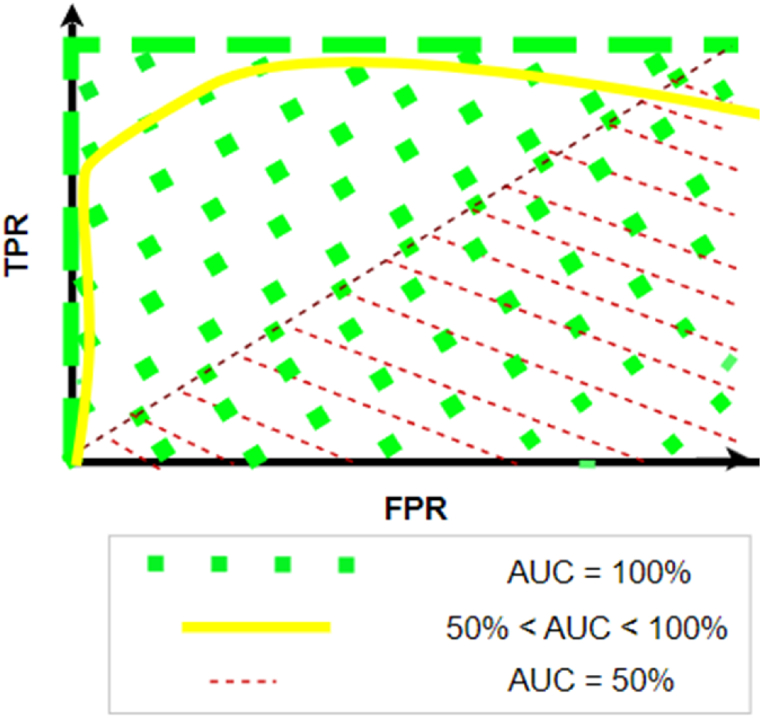
– RoC with AUC for three different classifiers.

Next is AUC of the Receiver Operating Characteristic Curve. The ROC graph depicts (Fig. 4) how a classifier performs under different thresholds of False Positive Rate (FPR) on the x-axis and True Positive Rate (TPR) on the y-axis.

Where TPR, the True Positive Rate is computed as:

$$TPR=\frac{TP}{TP+FN}$$

And FPR, the False Positive Rate is computed as:

$$FPR=\frac{FP}{FP+TN}$$

AUC summarizes the area under the ROC. Higher the AUC better the model. In Fig. 4 we can see ROC of three different classifiers. The region indicated by green dots is the 100 % Area Under the Curvie of the ROC. This indicates a perfect classifier. The region covered by red dotted lines is the ROC of a classifier which makes random guess, i.e. it cannot discriminate between the two classes. Ideally, a classifier with AUC close to 100 % is a good classifier, represented by yellow solid curve in this case.

Both our test datasets are imbalanced and hence it is important to look at all the scores (F1, ROC, and AuC) for a comprehensive picture.

F1 may not provide a comprehensive and balanced assessment of a classifier, especially when there is a class imbalance in a dataset. It can be observed from the F1 score equation, that higher F1 values maybe produced even if the performance of minority class is poor as it does not take true negatives into account. MCC incorporates all four elements of the confusion matrix (true positives, true negatives, false positives, and false negatives), ensuring a comprehensive evaluation.

There have been several studies that demonstrate MCC and ROC-AUC as a more reliable metric over other metrics like accuracy and F1-score [[10], [11], [12],^41^].

We therefore focus on F1 score, MCC, and AUC score for a comprehensive evaluation of our classifiers and the DeepMoji's fine-tuned model.

### 3.9 Experimental results

In this section we will showcase the results of experiments for different models created. All the experiments were conducted for five different runs. The results reported in Table 2, Table 3 are averaged over five runs.

**Table 2:** Results of DeepMoji pre-trained model finetuned on SarcOji dataset.

Model	DeepMoji (Fine tuning approach)	SarcOjiTest1			SarcOjiTest2		
		F1	MCC	Roc	F1	MCC	ROC-AUC
PlainText	N.A.						
TextWithEmojis	Chain-thaw	52.05	−0.1	45.7	26.83	−0.13	42.35
	Last	**68.14**	**0.11**	**53.53**	37.68	−0.01	49.41
	Full	65.26	−0.02	50.23	**40.09**	**0.05**	**52.97**
	New	60.91	−0.01	49.35	31.56	−0.1	47.44
TextMaxEmoji	Chain-thaw	50.25	−0.08	46.03	26.95	−0.15	41.29
	Last	**69**	**0.1**	**52.45**	**39.31**	**0**	**50.27**
	Full	48.64	−0.08	45.8	24.96	−0.15	41.23
	New	53.62	−0.06	47.18	27.92	−0.16	41.17

**Table 3:** – Proposed models’ performance.

Model	Architecture	SarcOjiTest1			SarcOjiTest2		
		F1	MCC	RoC	F1	MCC	ROC-AUC
PlainText	Vanilla Sequential	52.01	0.05	52.48	37	0.07	54.32
	LSTM	53.91	0.09	54.37	40.06	0.13	57.36
	BiLSTM	54.41	0.09	54.68	43.1	0.18	60.03
	Additive Attention	34.45	0.08	54.31	**45.71**	**0.04**	**52.22**
	Multiplicative Attention	**35.56**	**0.1**	**55.24**	28.15	0.02	50.93
TextWithEmojis	Vanilla Sequential	51.41	0.05	52.48	35.12	0.04	52.5
	LSTM	53.74	0.08	54.18	42.36	0.16	59.37
	BiLSTM	53.34	0.07	53.7	42.92	0.17	59.96
	Additive Attention	43.07	0.18	60.32	**52.72**	**0.11**	**54.95**
	Multiplicative Attention	**50.19**	**0.33**	**66.83**	45.09	0.09	54
TextMaxEmoji	Vanilla Sequential	51.81	0.05	52.13	37	0.07	54.32
	LSTM	55.13	0.11	55.25	40.06	0.13	57.36
	BiLSTM	54.61	0.1	54.96	43.1	**0.18**	**60.03**
	Additive Attention	44.94	0.24	62.5	**54.95**	0.12	56.1
	Multiplicative Attention	**52.32**	**0.36**	**68.29**	50.02	0.14	56.57

In Table 2 we can see the results of DeepMoji's pretrained model finetuned on SarcOji dataset using the different transfer learning approaches discussed in the previous section. Since DeepMoji architecture and training rely on emojis for emotional cues it won't be effective for the PlainText model. Hence, we test DeepMoji's fine-tuned model for TextWithEmojis and TextMaxEmoji models.

We observe high F1-scores for ‘Last’ and ‘Full’ approaches in both the models. SarcOjiTest1 has a very high F1-score of 68.14 % and 69 % for the ‘Last’ fine-tuning approach. We do not observe very high F1-scores for SarcOjiTest2. But the performance of the classifiers is extremely poor when we look at the MCC and ROC-AUC scores with all the classifiers showing inability to distinguish between positive and negative classes.

Table 3 records the results of the three models we tested across different architectures proposed in this work.

From these tables we note that the addition of emojis to text (TextWithEmojis and TextMaxEmoji models) helps improve in detection. We observe an increase in F1-scores, MCC, and ROC-AUC when we add emojis to plain text. The TextMaxEmoji model achieved the highest F1, MCC, and ROC-AUC scores for both the datasets. However, the F1, MCC, and ROC-AUC scores were very low for SarcOjiTest2 as compared to SarcOjiTest1.

Based on these observations we can conclude that the TextMaxEmoji model outperformed PlainText and TextWithEmojis models on all the three metrics. This means MaxEmoji or the most frequent emoji in text is an indicator of sarcasm.

## 4 Discussion

In general, it is evident from Table 2, Table 3 that addition of emojis to text (TextWithEmojis and TextMaxEmoji models) helps improve classification performance as compared to providing only text input to the classifiers (PlainText model) when we look at the MCC and ROC-AUC scores.

### 4.1 Performance on SarcOjiTest1

**Fig. 5:**
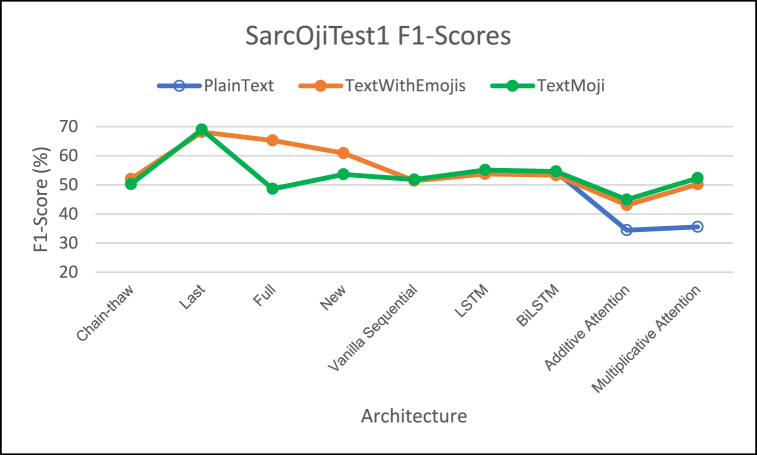
– Comparison of F1 Scores for different architectures on SarcOjiTest1.

**Fig. 6:**
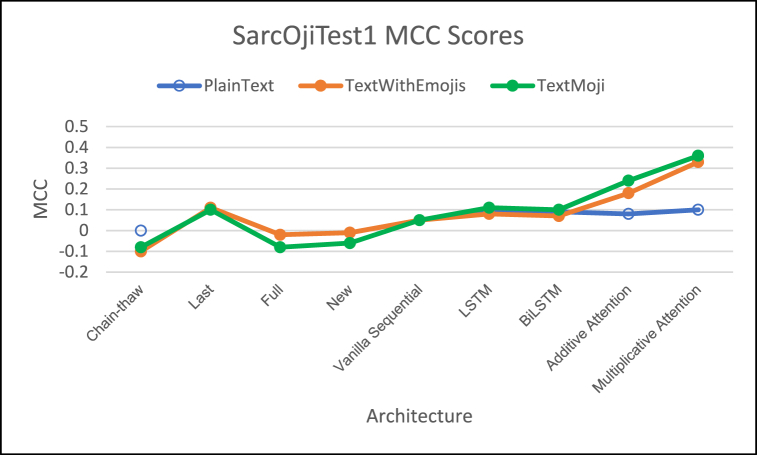
– Comparison of MCC Scores for different architectures on SarcOjiTest1.

**Fig. 7:**
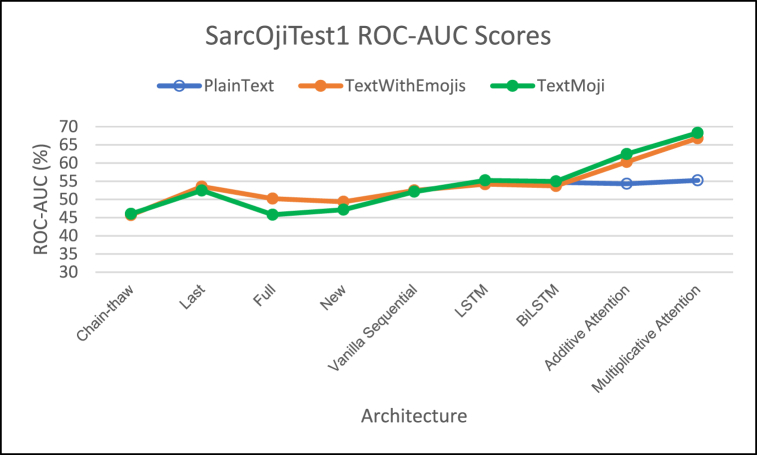
– Comparison of ROC-AUC Scores for different architectures on SarcOjiTest1.

Performance of different architectures on SarcOjiTest1 is provided in Fig. 5 (F1-Score comparison), Fig. 6 (MCC score comparison), Fig. 7 (ROC-AUC score comparison). We have not included the PlainText model for DeepMoji fine-tuned approaches as the PlainText model was not applicable for DeepMoji.

For SarcOjiTest1, the DeepMoji fine-tuning approaches achieve higher F1 scores than the proposed architectures in this work.

We note that MCC for DeepMoji approaches is much lower than the architectures proposed in this study. The highest MCC score is observed for the TextMaxEmoji model across all deep-learning networks with the multiplicative attention network.

The ROC-AUC scores for the attention networks are higher with TextMaxEmoji model having a slightly higher ROC-AUC scores as compared to TextWithEmojis model. The multiplicative attention architecture achieves the highest ROC-AUC scores for both emoji based models proposed in this work. But the ROC-AUC scores for the DeepMoji approaches is much lower than the architectures created for this study.

While addition of emojis certainly improved performance of the classifiers, the attention mechanisms prove to be better classifiers as compared to the other deep-learning architectures giving highest F1, ROC-AUC, and MCC scores for both the emoji-based models proposed in this work.

Employing attention networks on the proposed models (TextWithEmojis and TextMaxEmoji) fared better across all metrics for SarcOjiTest1 dataset. We can thus conclude that the addition of emojis to text helped improve sarcasm classification.

### 4.2 Performance on SarcOjiTest2

**Fig. 8:**
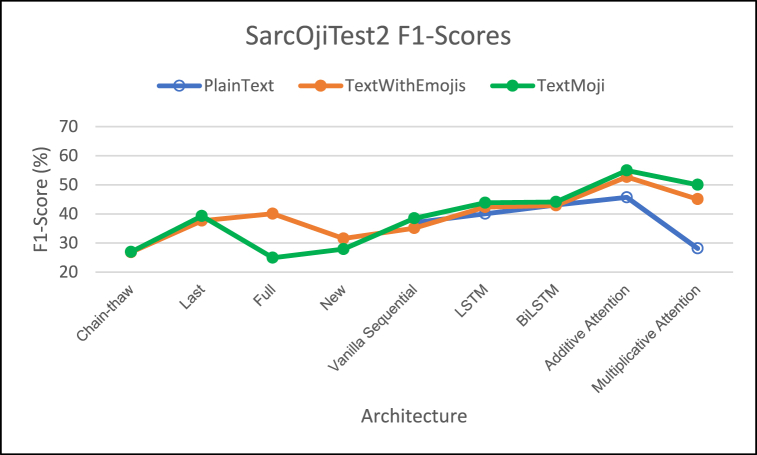
– Comparison of F1 Scores for different architectures on SarcOjiTest2.

**Fig. 9:**
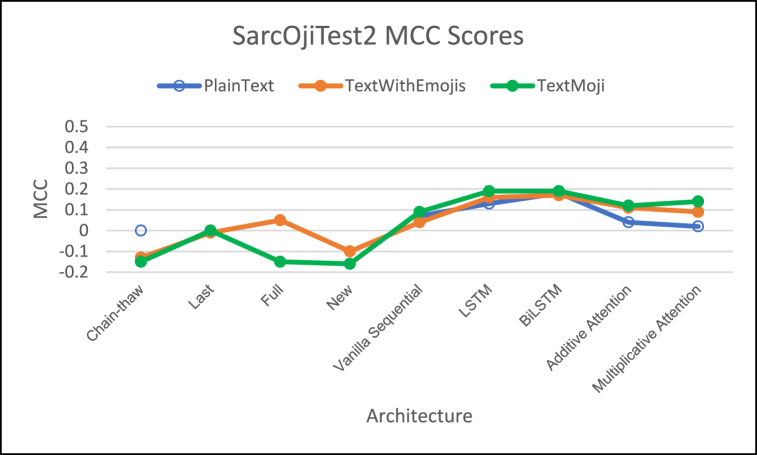
– Comparison of MCC Scores for different architectures on SarcOjiTest2.

**Fig. 10:**
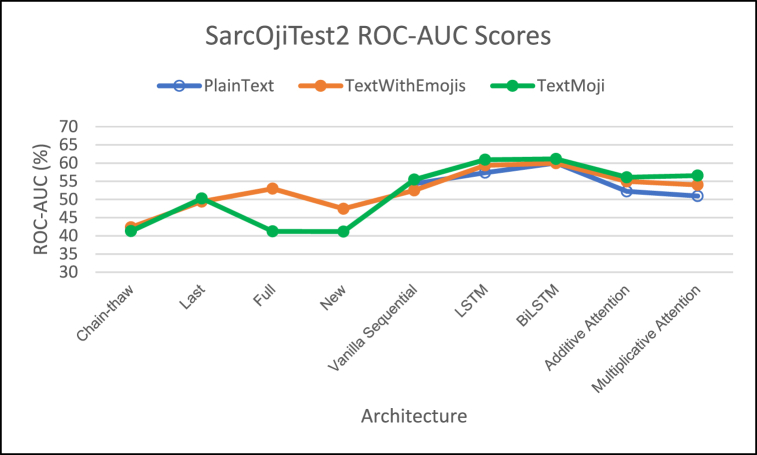
– Comparison of ROC-AUC Scores for different architectures on SarcOjiTest2.

Fig. 8, Fig. 9, Fig. 10 demonstrate the comparison of F1-Score, MCC Score, and ROC-AUC Score respectively.

For SarcOjiTest2 we observe lower F1 scores on all the architectures as compared to SarcOjiTest1. We observe that the proposed models TextWithEmojis and TextMaxEmoji achieve higher F1-scores as compared to the DeepMoji fine-tuned models.

Looking at the MCC scores, we observe that the proposed model/architecture combinations outperformed the DeepMoji fine-tuning approaches. The MCC scores for TextMaxEmoji is highest when we employ the attention architectures and is slightly better than the TextWithEmojis model tested with DeepMoji's last fine-tuning approach. Attention mechanisms outperform other architectures for this test set too.

We observe high ROC-AUC scores for LSTM and Bi-LSTM networks for this test set. In general, the PlainText models have lower ROC-AUC scores (except for PlainText Bi-LSTM model) and ROC-AUC increase with addition of emojis to text, with highest ROC-AUC scores for TextMaxEmoji model.

The emoji-based models TextWithEmojis and TextMaxEmoji model had higher F1, MCC, and ROC-AUC scores than the PlainText model but these scores were much lower as compared to those observed on SarcOjiTest1.

### 4.3 Comparison with DeepMoji and the use of F1-score as metric for classifier performance

Take a look at Table 4 that compares the highest performing models for DeepMoji and proposed work.

**Table 4:**
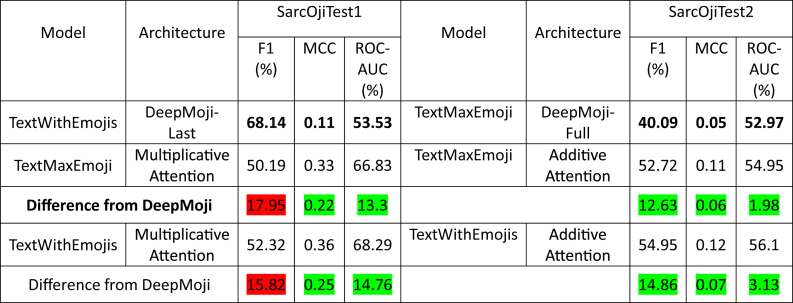
Comparison of proposed model/architecture performance with DeepMoji.

For simplicity we pick model/architecture combinations with highest F1-scores. We add difference from DeepMoji to the respective cell.

In Table 4 the figures highlighted in red depict a reduction in metric score from DeepMoji while the ones in green depict a gain in the respective metric score.

While DeepMoji approaches achieved very high F1-score, MCC score was ≤0.11 (in some cases negative) and ROC-AUC score was ≤53.53% and in some cases less than 50%. A score of 50% ROC-AUC or 0 MCC indicates that the model is making random predictions. A negative MCC or a ROC-AUC score < 50% means that the model moving in the direction of predicting the opposite of the labels it is supposed to predict.

Our proposed models outperformed DeepMoji on SarcOjiTest1 on MCC and ROC-AUC scores with ≥0.33 MCC and ≥66.83% ROC-AUC score for attention mechanisms. This indicates that the proposed model/architectures have stronger distinguishing capabilities as compared to DeepMoji.

It is evident from this comparison that even though DeepMoji attains a high F1-score for SarcOjiTest1 it fails at MCC and ROC-AUC scores. For SarcOjiTest2 we observe a gain in all the three metrics F1, MCC, and ROC-AUC scores.

We can thus conclude that F1 should not be used as a sole metric for comparison of classifiers.

### 4.4 Conclusion and future work

Based on this study we conclude that emojis are a powerful modality that can help classifiers discern sarcasm. The best performance is observed with TextMaxEmoji model where we remove all the emojis from the text and append MaxEmoji at the end. We also observe that the attention mechanisms additive attention and self-attention mechanism can significantly improve classifier performance when we employ emoji for sarcasm detection task. The improvement in MCC score and ROC-AUC scores for the proposed models is observed on two unseen datasets hence we can say that the proposed models have a better generalization capability as compared to DeepMoji. In general, all the classifiers perform better on SarcOjiTest1 dataset as compared to the performance on SarcOjiTest2 dataset.

Another interesting learning from this study is that while comparing classifiers F1 alone should not be considered as the only measure. While most research studies only report F1-score, it may not give us a complete view of a classifier's performance and it may even be misleading especially when the datasets are imbalanced. Thus, other metrics like MCC and AUC need to be compared too, as they help provide a comprehensive picture of how good a classifier is.

With this work we have successfully answered the three research questions we posed.

1. The experiments established that emojis play a crucial role in discerning sarcasm even in the absence of other modalities. We observed a significant improvement in all the three metrics for TextWithEmojis and TextMaxEmoji model as compared to the PlainText model.
2. Addition of the MaxEmoji to text helped in improving in sarcasm classification for both the test sets. A significant improvement in all the three metrics was observed for this model as compared to PlainText, TextWithEmojis, and DeepMoji models for the test sets used. This could indicate that the repeated use of an emoji is an indicator of the user's intent in the text.
3. We could also observe that a single metric like F1 or accuracy may not be a sole indicator of a good classification capability of a classifier. This was observed when we compared classifier performance of the benchmark DeepMoji and the proposed models on three different metrics. While DeepMoji gave a better F1-score it fared poorly in MCC and ROC-AUC scores for both our test sets. Hence, we conclude that to investigate performance of classifiers a combination of metrics must be used. In this set of experiments, we used F1, MCC, and ROC-AUC score for a comprehensive evaluation of classifiers.

In future we aim to improve the performance of the proposed models especially on SarcoOjiTest2 which is randomly derived from texts on Twitter (now X).

## Funding

NA.

## Ethics

The study design, data presentation and writing style comply with journal's Editorial Policies.

## Data availability

The training and test datasets used for the experiments in this work are available at https://github.com/VanditaGroverKapila/SarcOji.

## CRediT authorship contribution statement

**Vandita Grover:** Writing – original draft, Visualization, Validation, Methodology, Investigation, Formal analysis, Data curation, Conceptualization. **Hema Banati:** Writing – review & editing, Supervision, Project administration, Methodology, Conceptualization.

## Declaration of competing interest

The authors declare that they have no known competing financial interests or personal relationships that could have appeared to influence the work reported in this paper.
